# Comparing Two Methods of Aortic Annulus Computed Tomography Measurements: A Retrospective Teleradiology Saudi Study

**DOI:** 10.7759/cureus.51564

**Published:** 2024-01-03

**Authors:** Amr M Ajlan

**Affiliations:** 1 Radiology Department, Faculty of Medicine, King Abdulaziz University Hospital, Jeddah, SAU; 2 Radiology Department, Diagnostics Elite Teleradiology, Jeddah, SAU

**Keywords:** computed tomography, cardiac imaging, aortic valve, tavi, transcatheter aortic valve implantation

## Abstract

Objectives

The objective of this study was to determine the overall mean size of the aortic valve annulus and to compare two distinct methods of quantifying aortic valve annulus dimensions using computed tomography (CT) in pre-transcatheter aortic valve implantation (TAVI) Saudi teleradiology cohort.

Materials and methods

This retrospective cohort study, conducted from December 2019 to September 2023, included 31 patients identified using "TAVI" in our teleradiology picture archiving and communication system. CT examinations followed standardized protocols. Three experienced radiologists assessed the aortic valve annulus, measuring maximum, minimum, and mean transverse diameters, area, and area-derived diameter. The statistical analysis involved calculating mean values and standard deviations and conducting t-tests to compare measurement methods.

Results

The study cohort had an average age of 73.35 ± 8.55 years, with 67.74% males. No significant age difference was observed between genders (p = 0.8421). Aortic valve annulus measurements showed the mean transverse diameter to be 22.51 ± 2.04 mm and the area-derived diameter at 22.83 ± 1.99 mm, with no significant difference between these methods (p = 0.53). Additional parameters included the maximum transverse measurement (25.78 ± 2.92 mm), minimum transverse measurement (19.23 ± 2.31 mm), and area (4.12 ± 0.72 cm²).

Conclusion

This study employed mean and area-derived diameter methods to evaluate the average size of the aortic valve annulus within a Saudi teleradiology cohort. The average sizes determined were 22.51 mm and 22.83 mm for the mean and area-derived diameter methods, respectively. The lack of a statistically significant difference between these two methods suggests their comparable efficacy in assessing aortic valve annulus size in this cohort.

## Introduction

Transcatheter aortic valve implantation (TAVI) represents a pivotal advancement in cardiac interventions, introduced in recent years as a revolutionary approach to managing critical aortic valve stenosis. Historically, this condition necessitated conventional surgical intervention, which posed significant hazards for high-risk patients. TAVI has emerged as a viable and less invasive alternative, specifically designed for those who are deemed unsuitable for traditional surgery due to elevated procedural risks. Remarkably, the success of TAVI has not been limited to high-risk cohorts; it has progressively been extended to encompass patients with intermediate and lower risk profiles. The outcomes of TAVI in these expanded patient groups have been promising, demonstrating efficacy and safety profiles comparable to those of conventional surgical valve replacement. This broadening of indications signifies a significant shift in the therapeutic landscape for aortic valve stenosis, offering a spectrum of patients a safer and equally effective treatment option [1].

Computed tomography (CT) has become a cornerstone in the preoperative assessment of patients undergoing TAVI, primarily attributed to its superiority in evaluating critical anatomical structures [2,3]. CT excels in delineating the aortic valve annulus and assessing the aortoiliofemoral access pathway, which is essential for guiding the TAVI procedure. The precise imaging capabilities of CT allow for a detailed understanding of patient-specific anatomy, which is vital for the successful planning and execution of TAVI [4].

In this study, we aim to utilize CT imaging to understand the aortic valve annulus dimensions in a Saudi population, using a teleradiology cohort to determine the average annulus size. We also compare two annulus assessment methods, the mean transverse diameter, and the area-derived diameter.

## Materials and methods

Subjects

The retrospective cohort was identified by querying the term "TAVI" within radiology reports from the picture archiving and communication system (PACS; PaxeraUltima, version 6.0.2.6, PaxeraHealth, Newton, MA, USA) from December 3, 2019, to September 14, 2023. From an initial pool of 40 identified cases, 31 patients were included in the final analysis, after excluding one case for motion artifact, one case for being a post-TAVI assessment, and seven cases for insufficient information. Patients were assessed for age and gender. An institutional review board was granted, and the consent form was weaved.

Image analysis

CT examinations were conducted per standardized protocols on various multidetector CT scanners. The technical performance of the CT scans and the subsequent aortic valve annular measurements adhered to established standard practices [4-6]. The measurements were conducted by a team of three radiologists with six to fourteen years of experience in cardiac CT imaging. The assessed aortic valve annulus parameters included maximum transverse measurement, minimum transverse measurement, mean transverse diameter, area, and area-derived diameter. All measurements, except for the area, were recorded in millimeters (mm); the area was quantified in square centimeters (cm²).

Statistical analysis

The statistical analysis was facilitated by ChatGPT, an advanced language model AI, version 4, developed by OpenAI, based in San Francisco, United States. The software computed mean values and standard deviations (SD) and conducted t-tests to assess the statistical significance between the two methods of annulus measurement. To ensure the validity of the computational analysis, all results were independently verified by a human researcher.

## Results

In this cohort, comprising 31 patients undergoing pre-TAVI evaluation, the mean age was 73.35 years, with an SD of 8.55 years, reflecting a diverse age range. The group consisted predominantly of male patients, totaling 21 (67.74%), while female patients constituted 32.26% with 10 individuals. Analyzing the age distribution more closely, male patients had an average age of 73.57 ± 8.95 years, marginally higher than the female patients, whose average age was 72.90 ± 8.10 years. However, a t-test to compare the ages across genders indicated no statistically significant difference (p-value = 0.8421), suggesting age distribution was relatively uniform between male and female patients.

**Figure 1:**
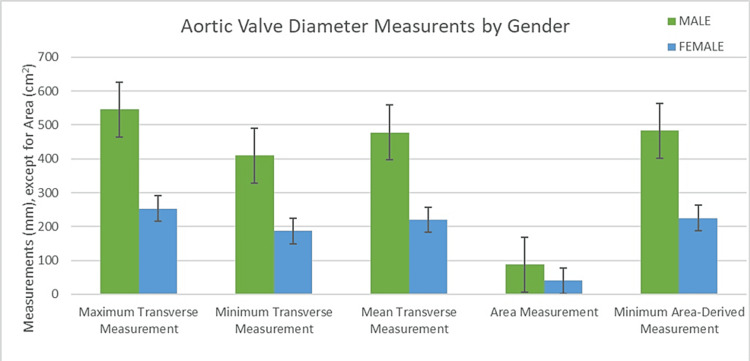
Aortic valve annulus measurements by gender

**Figure 2:**
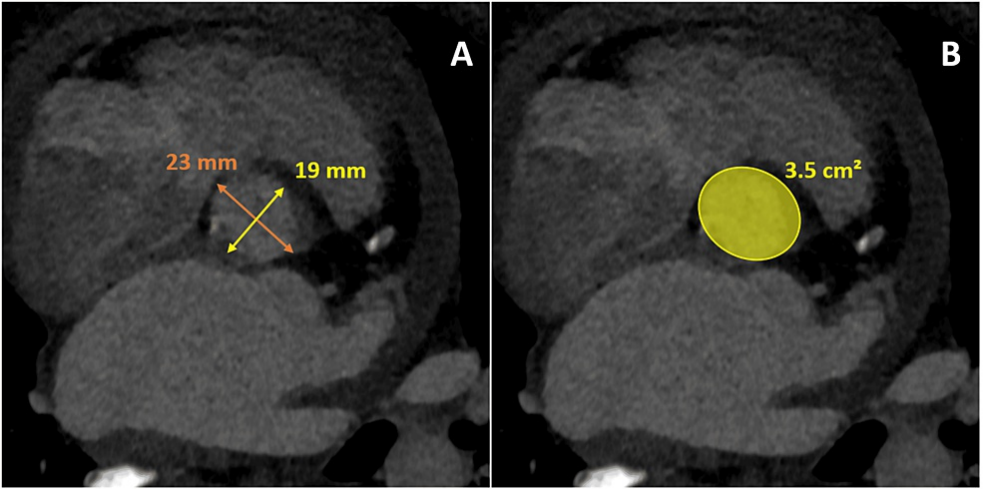
An example of aortic valve annulus measurement on CT in a 63-year-old female patient with severe aortic stenosis. Image A shows that the maximum transverse diameter was 23 mm and the minimum transverse diameter was 19 mm, resulting in a mean transverse diameter of 21 mm. Image B shows that the annulus area of the aortic valve was 3.5 mm, yielding an area-derived diameter of 21.1 mm.

Detailed in Table 1 are the descriptive statistics for the aortic valve annulus measurements, encompassing both maximum and minimum transverse measurements alongside the mean and area-derived diameters. The maximum transverse measurement of the aortic valve annulus across the cohort was 25.78 ± 2.92 mm. In comparison, the minimum transverse measurement averaged 19.23 ± 2.31 mm. The annulus area was calculated to be 4.12 ± 0.72 cm². Further detailed analysis of aortic valve annulus measurements provided nuanced insights. The mean transverse diameter was 22.51 ± 2.04 mm across the cohort. In contrast, the area-derived diameter averaged slightly higher at 22.83 ± 1.99 mm. A statistical analysis comparison between these two measurement methods revealed no significant difference (p-value = 0.53). Figure 1 is a bar chart comparing the various measures of the aortic valve annulus in both genders. On average, male patients had slightly larger aortic valve measurements regarding maximum transverse measurement, minimum transverse measurement, mean diameter, and area. The aortic valve annulus area-derived diameter was also slightly higher in males than in females. Figure 2 demonstrates an example of the maximum transverse diameter, minimum transverse diameter, and area measures of the aortic valve annulus.

**Table 1:** Descriptive statistics for aortic valve annulus measurements

Parameter	Average (mm)	Standard Deviation (mm)
Maximum Transverse Measurement	25.78	2.92
Minimum Transverse Measurement	19.23	2.31
Mean Transverse Diameter	22.51	2.04
Area (cm²)	4.12	0.72
Area-Derived Diameter	22.83	1.99

## Discussion

In this study of a group of Saudi patients gathered from a teleradiology cohort, the mean transverse diameter of the aortic valve annulus was 22.51 ± 2.04 mm. In contrast, our measured area-derived diameter averaged 22.83 ±1.99 mm. In general, such annular measurements are smaller on average compared to other studies assessing the annular diameter of the aortic valve on CT. For example, in a survey by Dashkevich et al., the mean transverse diameter measured on CT was 24.5 ± 2.6 mm [7]. In another study by Choe et al., the aortic annulus mean transverse diameter was 25.6 ± 3.1 2 mm, and the area-derived diameter was 25.2 ± 3.1 [8].

In the context of sizing the aortic valve annulus for CT scans, a combined approach utilizing the mean transverse diameter and area-derived diameters is recommended over relying solely on the mean transverse diameter [6]. Consequently, this study aims to determine whether there are significant differences in accuracy or clinical outcomes when comparing these two measurement methodologies. A few studies have explored the implications of over- or under-sizing aortic valve annulus measurements, employing various methods. However, to our knowledge, there is a gap in the literature regarding head-to-head comparisons of the two commonly used measurement techniques: the mean transverse diameter and area-derived diameters [9,10]. Our current study showed no statistically significant difference between the two aortic valve annulus measurement methods.

The relatively small sample size in this study constitutes a potential limitation. Such a limitation is anticipated in a private hospital setting, where TAVI procedures are less frequently performed. Nevertheless, utilizing data from a teleradiology service facilitated a more comprehensive aggregation of patient data from several hospitals, thereby enhancing the study's catchment. Another factor to consider is the potential interobserver variability among the three radiologists, which might have influenced the precision of the annular measurements. Finally, variations in the CT scanning acquisition phase, specifically whether performed during systole or diastole, could have affected the accuracy of the annulus size measurements [11].

## Conclusions

In summary, this investigation offers insights into the aortic valve annulus assessment using CT imaging within a Saudi teleradiology context, particularly in evaluating patients for TAVI. By juxtaposing two measurement methodologies (i.e., mean transverse and the area-derived diameters), our study underscores their potential comparable efficacy in clinical practice. These findings contribute to the existing knowledge in cardiac imaging and emphasize the importance of methodological versatility in preoperative evaluations for TAVI. However, it is crucial to acknowledge that this study represents an initial exploration in a relatively limited cohort. Therefore, future research involving larger patient populations must further validate and refine these measurement approaches. Expanding the study scope will enhance the reliability of conclusions and impact the optimization of patient selection and treatment strategies in TAVI, thereby improving clinical outcomes.
